# Association among pre-pregnancy body mass index, gestational weight gain and neonatal birth weight: a prospective cohort study in China

**DOI:** 10.1186/s12884-020-03323-x

**Published:** 2020-11-12

**Authors:** Yawen Wang, Haihui Ma, Yahui Feng, Yongle Zhan, Sansan Wu, Shuya Cai, Yingjie Shi, Yunli Chen, Liangkun Ma, Yu Jiang

**Affiliations:** 1 School of Population Medicine and Public Health, Chinese Academy of Medical Sciences/Peking Union Medical College, Beijing, 100730 China; 2 The Jockey Club School of Public Health and Primary Care, The Chinese University of Hong Kong, Hongkong, Hong Kong, China; 3 Department of obstetrics, Tongzhou Mater&Child health hospital of Beijing, Yuqiaozhong Road, Tongzhou District, Beijing, 101100 China; 4 Department of Obstetrics, Peking Union Medical College Hospital, Chinese Academy of Medical Sciences/Peking Union Medical College, No.1, Shuaifuyuan Wangfujing Dongcheng District, Beijing, 100730 China

**Keywords:** Body mass index, Gestational weight gain, Small for gestational age, Large for gestational age

## Abstract

**Background:**

This study aims to explore the relationships between pre-pregnancy body mass index (BMI), gestational weight gain (GWG), rate of GWG during the second and third trimesters (GWG_rate_) and birth weight among Chinese women.

**Methods:**

Women were enrolled by 24 hospitals in 15 different provinces in mainland China from July 25th, 2017 to 26 November 2018. Pre-pregnancy BMI, GWG and GWG_rate_ were calculated and divided in to different groups. The multinomial logistic regression model and restrictive cubic spline model were used to explore the relationships.

**Results:**

Of the 3585 participants, women who were underweight, had insufficient GWG or GWG_rate_ had 1.853-, 1850- or 1.524-fold higher risks for delivering small-for-gestational-age (SGA) infant compared with women who had normal BMI, sufficient GWG or GWG_rate_. Women who were overweight/obese, had excessive GWG or GWG_rate_ had 1.996-, 1676- or 1.673-fold higher risks for delivering large-for-gestational-age (LGA) infant. The effects of GWG and GWG_rate_ on birth weight varied by pre-pregnancy BMI statuses. Dose-response analysis demonstrated L-shaped and S-shaped relationships between pre-pregnancy BMI, GWG, GWG_rate_ and neonatal birth weight.

**Conclusions:**

Pre-pregnancy BMI, GWG or GWG_rate_ were associated with neonatal birth weight among Chinese women. Both body weight before and during pregnancy should be maintained within the recommendations to prevent abnormal birth weight.

## Background

Since the 1980s, the overweight and obesity rates have doubled during the past four decades in more than 70 countries worldwide, and the rates are still increasing [1]. The same trend have been observed among Chinese adults: the overweight and obesity rates among this population were 20 and 7.1%, respectively, according to a national survey conducted in 2002 [2]. For women of childbearing age, pre-pregnancy body mass index (BMI) is regarded as an important predictor of pregnancy outcomes since abnormal BMI has been verified to be related to a series of adverse maternal and neonatal complications [3–5]. However, the incidence of pre-pregnancy overweight and obesity has increased significantly around the world and has become an urgent public health problem. Gestational weight gain (GWG) is another factor that impacts fetal development, and insufficient or excessive GWG mostly results in restricted or over development. Asian women have been shown to have the highest prevalence of insufficient GWG (USA 21%, Europe 18% and Asia 31%), while more than half of American women have excessive GWG (USA 51%, Europe 51% and Asia 31%) [6].

Although GWG is commonly used to evaluate fetal development, it has some limitations. Weight gain has different effects on fetal development in different trimesters, and total GWG failed to show trimester-specific weight change during pregnancy. A large number of clinical studies have demonstrated that women’s weight gain during the 2nd and 3rd trimester (GWG_rate_), which is also the period of maximal growth and weight gain for fetuses, is significantly associated with newborn weight while weight gain in the 1st trimester mostly affects overweight or obesity risk in the offspring’s childhood [7]. Thus, GWG_rate_ is preferable for exploring the relationship between trimester-specific weight change and infant birth weight even though the data collection is complicated. The overall prevalence of insufficient and excessive GWG_rate_ varies in different regions [8, 9]. Some studies showed that 12.5 and 57.9% of Chinese women had insufficient and excessive GWG_rate_ respectively in 2013 [10], but the updated incidence rate is unclear.

Most studies have shown that pre-pregnancy underweight and insufficient GWG are associated with a higher risk of having small for gestational age (SGA) infants but a lower risk of delivering large for gestational age (LGA) infants [11]. Similarly, pre-pregnancy overweight/obese and excessive GWG are associated with higher LGA risk but lower SGA risk despite of few studies that have inconsistent results [12–14]. However, some research indicates that the relationship between pre-pregnancy BMI, GWG and infant birth weight varies among different races of women, and whether the relationship is the same among Chinese women is not clear [15]. In addition, most research in China is province-based or city-based and lacks representativeness. As for the association between GWG_rate_ and birth weight, relevant studies are even mere, and the results are also inconsistent [8, 16]. Furthermore, few studies have explored the dose-response relationship between women’s body weight before or during pregnancy and infant birth weight. Hence, conducting more related studies and making the results more comprehensive is meaningful.

This study aimed to evaluate the prevalence of pre-pregnancy BMI, GWG and GWG_rate_ in the 2nd and 3rd trimester, and to explore their associations with abnormal neonatal birth weight (i.e. SGA and LGA) among Chinese women, as well as the dose-response relationship between women’s weight status before or during pregnancy and abnormal birth weight risks.

## Methods

### Study setting and study population

The Chinese Pregnant Women Cohort Study (CPWCS) is a multicenter, prospective cohort study focusing on antenatal women and their neonates. Twenty-four hospitals [see Table S1 in Additional file] distributed in 15 provinces in China were selected as the center sites through the comprehensive consideration of geography and economy. Pregnant women visiting anyone of 24 cooperated hospitals for their first trimester antenatal clinic with a gestational age of 5–13 weeks were enrolled. The study period of this cohort study was from 25 July 2017 to 26 November 2018 and the inclusion criteria were as follows: (1) 16 years old or above; (2) gestational age at 5–13 weeks (during the first trimester); (3) able to complete the questionnaires; (4) permanent residents (dwell in the local site for over 6 months) in the study locations; and (5) willing to sign the written informed consent form. In addition, women enrolled in this study must had single live birth after 28 gestational weeks. Other exclusion criteria included: less than 16 years of age; temporary residence; serious chronic diseases; psychosis; twin or multiple pregnancies; non-single live birth; and less than 28 gestational weeks.

### Data collection

#### Original data

Participants were required to complete an electronic self-designed questionnaire in their first trimester and were followed up three times: in the second trimester, in the third trimester, and 42 days postpartum. Information provided by respondents includes basic sociodemographic characteristics, lifestyle behaviors, complications in each trimester and pregnancy outcomes. The pre-pregnancy weight and height of each respondent were self-reported when women were enrolled in this study, and pre-pregnancy BMI was assessed. Body weight at the beginning of the second trimester and at delivery were also collected, and the weight difference was GWG. Some important variables, such as pre-pregnancy body weight and weight at delivery, height, pregnancy complications, gestational age and pregnancy outcome were also checked with data recorded in the hospital information system (HIS) to maintain data quality.

#### Classification of pre-pregnancy BMI, GWG and GWG_rate_

Pre-pregnancy BMI (weight(kg)/ height (m)^2^) was calculated by respondent’s height and pre-pregnancy weight and was categorized into four groups according to standard WHO criteria: underweight (BMI < 18.5 kg/m^2^), normal-weight (18.5 kg/m^2^ ≤ BMI ≤ 24.9 kg/m^2^), overweight (25.0 kg/m^2^ ≤ BMI ≤ 29.9 kg/m^2^) and obese (BMI ≥ 30.0 kg/m^2^) [17].

GWG was defined as the difference in weight at delivery and weight before pregnancy. According to the IOM recommendation, for pre-pregnancy underweight, normal weight, overweight and obese women, the appropriate GWGs are 12.5–18 kg, 11.5–16 kg, 7–11.5 kg and 5–9 kg, respectively. Women with GWG below the recommended range were defined as having insufficient GWG, and those with GWG above the appropriate range were defined as having excessive GWG [18].

GWG_rate_ was calculated as (the difference of weight at delivery and weight at the beginning of 2nd trimester)/(gestational age at delivery-13), whereas 13 was the cutoff value of the 1st and 2nd trimesters. According to IOM, for pre-pregnancy underweight, normal weight, overweight and obese women, the appropriate GWG_rate_ ranges are 0.44–0.58 kg/w, 0.35–0.50 kg/w, 0.23–0.33 kg/w and 0.17–0.27 kg/w, respectively [18]. Women with a GWG_rate_ below the recommended range were defined as having insufficient GWG_rate_, and those with GWG_rate_ above the appropriate range were defined as having excessive GWG_rate_.

#### Definition of neonatal birth weight

The main outcomes of this study were SGA and LGA. Birth weights below the 10th or above the 90th percentile, respectively, for the same gestational age by sex were defined as SGA and LGA according to the Chinese neonatal birth weight curve [19].

#### Covariate assessments

In this study, covariates included sociodemographic characteristics (i.e., maternal age, ethnicity, residential areas, educational level, occupation and annual household income), lifestyle behaviors (i.e., smoking and alcohol consumption), and clinical characteristics (gravidity, parity, gestational diabetes mellitus (GDM) and gestational hypertension). Gestational age was calculated as the difference in date from the last menstrual period to delivery. Alcohol consumption was defined as consuming any alcoholic beverage more than once per month. GDM was defined as meeting one or more of the following criteria: fasting plasma glucose ≥5.1 mmol/L, 1 h plasma glucose levels ≥10.0 mmol/L, 2 h plasma glucose levels ≥8.5 mmol/L after overnight fasting with a 75 g glucose load at 24–28 gestational weeks according to the diagnostic criteria amended by WHO in 2013 [20].

### Statistical analysis

Qualitative variables were described as the mean values and standard deviations while quantitative variables were described as frequencies and percentages. The chi-square test and Kruskal-Wallis test were used for univariable analyses. Multinomial logistic regression models were conducted to explore the relationship between pre-pregnancy BMI, GWG, GWG_rare_, and birth weight and the results are shown as odds ratios (ORs) and 95% confidence intervals (CIs). Dose-response relationships were explored by restricted cubic spline (RCS) logistic regression models. Three knots were located at the 25th, 50th and 75th percentiles of the distribution of each continuous dependent variable. RCS analysis was conducted by SAS 9.4 software with the RCS_Reg macro. All *P* values in this study were two-sided and *P* < 0.05 was regarded as a significant difference.

## Results

A total of 3585 women were enrolled and maternal characteristics presented by neonatal birth weight are shown in Table 1. The overall prevalence of SGA and LGA was 5.77 and 10.54%, respectively, among these respondents, and the average birth weight was 3321 g (SD: 453 g). Women who were younger and employed were more likely to have SGA infants, while older and unemployed women tended to have LGA infants (*P* < 0.05). SGA mothers were shown to have fewer gravidities and parities than LGA mothers. In addition, women who gave birth to SGA infants were less likely to have GDM but had higher risk of gestational hypertension compared with women in the LGA group (all *P* < 0.05).

**Table 1:** Maternal characteristics presented by neonatal birth weight [n(%)]

Items	Total *N* = 3585	SGA	AGA	LGA	χ^2^/H	P
		*N* = 207	*N* = 3000	*N* = 378		
Age					3.004	**0.048**
< 25	576 (16.07)	37 (17.87)	482 (16.07)	57 (15.08)		
25~	1804 (50.32)	113 (54.59)	1516 (50.53)	175 (46.30)		
30~	859 (23.96)	39 (18.84)	721 (24.03)	99 (26.19)		
35~	346 (9.65)	18 (8.70)	281 (9.37)	47 (12.43)		
Ethnicity					3.044	0.218
Han	3413 (95.20)	192 (92.75)	2859 (95.30)	362 (95.77)		
Minority	172 (4.80)	15 (7.25)	141 (4.70)	16 (4.23)		
Residential Areas					0.425	0.809
Urban	1594 (44.46)	90 (43.48)	1341 (44.70)	163 (43.12)		
Rural	1991 (55.37)	117 (56.52)	1659 (55.30)	215 (56.88)		
Educational level					10.422	0.108
Less than high school	508 (14.17)	21 (10.15)	418 (13.93)	69 (18.26)		
High school	765 (21.34)	42 (20.29)	648 (21.60)	75 (19.84)		
Bachelor	2082 (58.07)	133 (64.25)	1735 (57.84)	214 (56.61)		
Master or above	230 (6.42)	11 (5.31)	199 (6.63)	20 (5.29)		
Occupation					14.378	**0.026**
No	1064 (29.68)	42 (20.29)	908 (30.27)	114 (30.16)		
Yes	2521 (70.32)	165 (79.71)	2092 (69.73)	264 (69.84)		
Annual household Income (thousand)					3.628	0.459
< 70	959 (26.75)	44 (21.26)	816 (27.20)	99 (26.19)		
70~	1656 (46.19)	104 (50.24)	1375 (45.83)	177 (46.83)		
200~	970 (27.06)	59 (28.50)	809 (26.97)	102 (26.98)		
Smoking					0.881	0.644
No	3424 (95.51)	195 (94.20)	2868 (95.60)	361 (95.50)		
Yes	161 (4.49)	12 (5.80)	132 (4.40)	17 (4.50)		
Alcohol consumption					0.718	0.698
No	3325 (92.75)	189 (91.30)	2786 (92.87)	350 (92.60)		
Yes	260 (7.25)	18 (8.70)	214 (7.13)	28 (7.40)		
Gravidity					30.204	**< 0.001**
0	1085 (30.27)	96 (46.38)	883 (29.43)	106 (28.04)		
1	1192 (33.25)	53 (25.60)	1020 (34.00)	119 (31.48)		
2	755 (21.06)	35 (16.91)	627 (20.90)	93 (24.60)		
≥ 3	553 (15.42)	23 (11.11)	470 (15.67)	60 (15.88)		
Parity					8.236	**0.021**
0	2080 (58.02)	136 (65.70)	1733 (57.77)	211 (55.82)		
1	1292 (36.04)	63 (30.44)	1085 (36.17)	144 (38.10)		
2	145 (4.05)	4 (1.93)	123 (4.10)	18 (4.76)		
≥ 3	68 (1.89)	4 (1.93)	59 (1.96)	5 (1.32)		
GMD					6.122	**0.047**
No	3089 (86.17)	179 (86.47)	2600 (86.67)	310 (82.01)		
Yes	496 (13.83)	28 (13.53)	400 (13.33)	68 (17.99)		
Gestational Hypertension					8.611	**0.013**
No	3475 (96.93)	194 (93.72)	2917 (97.23)	364 (96.30)		
Yes	110 (3.07)	13 (6.28)	83 (2.77)	14 (3.70)		
Gestational Week	39.23 ± 1.44	39.15 ± 1.54	39.24 ± 1.43	39.13 ± 1.50	3.018	0.221

Table S2 to Table S4 [see Additional file] show the demographic and clinical characteristics of the study participants by pre-pregnancy BMI, GWG and GWG_rate_ categories. The prevalence of underweight, overweight and obesity before pregnancy was 14.17, 12.86 and 1.73%, respectively. According to the IOM recommendation, 24.69 and 33.78% of women had insufficient and excessive GWG, respectively. Table S4 [see Additional file] illustrates the prevalence of SGA and LAG by pre-pregnancy, GWG and GWG_rate_ categories. Approximately 26.86% of women showed insufficient GWG_rate_ while 46.95% had excessive GWG_rate_. The average birth weight of infants was associated with women’s pre-pregnancy BMI, GWG and GWG_rate_ (*P* < 0.001).

The multinomial logistic regression analysis results are shown in Table 2. Women who were underweight before pregnancy, had insufficient GWG or had insufficient GWG_rate_ were 1.9-,1,9- and 1.5-fold more likely to have SGA infants, respectively, compared with reference groups, and women who were overweight or obese before pregnancy, had excessive GWG or had excessive GWG_rate_ were 2.0-,1.7- and 1.7-fold more likely to have LGA infants, respectively. Additionally, protective effects were shown between excessive GWG_rate_ and SGA, as well as pre-pregnancy underweight, insufficient GWG and LGA.

**Table 2:** Adjusted OR for birth weight classified by pre-pregnancy BMI, GWG and GWG_rate_

Items	SGA		LGA	
	OR	95%CI	OR	95%CI
Pre-BMI ^a^				
Underweight	**1.853** ^c^	**1.316–2.610**	**0.575** ^d^	**0.383–0.864**
Normal weight	Ref.		Ref.	
Overweight/Obesity	0.576	0.328–1.010	**1.996** ^c^	**1.540–2.588**
GWG ^b^				
Insufficient	**1.850** ^c^	**1.309–2.616**	**0.601** ^d^	**0.399–0.903**
adequate	Ref.		Ref.	
Excessive	0.628	0.351–1.125	**1.676** ^c^	**1.279–2.194**
GWG_rate_^b^				
Insufficient	**1.524** ^d^	**1.068–2.174**	0.846	0.600–1.193
adequate	Ref.		Ref.	
Excessive	**0.668** ^d^	**0.458–0.973**	**1.673** ^c^	**1.274–2.196**

^a^ Adjusted for maternal age, occupation, gravidity, parity; ^b^ Adjusted for maternal age, occupation, gravidity, parity, GDM, gestational hypertension and pre-BMI; ^c^ *P* < 0.001; ^d^ *P* < 0.05

Chi-square analysis demonstrated the relationship between birth weight and jointed pre-pregnancy BMI and GWG/GWG_rate_ [see Table S5 to Table S7 in Additional file]. The prevalence of insufficient GWG in the pre-pregnancy underweight, normal weight and overweight/obesity groups was 28.54, 27.21 and 8.60%, respectively, and the prevalence of excessive GWG was 24.41, 30.58 and 58.51%, respectively, in each group. GWG and GWG_rate_ were associated with infant birth weight under different pre-pregnancy BMI levels (*P* < 0.05).

Stratified analysis results are shown in Table 3. Among the pre-pregnancy underweight, normal weight and overweight/obese groups, women with insufficient GWG were 3.0-, 1.2- and 6.7-fold more likely to have SGA infants, respectively. Excessive GWG among normal weight and overweight/obese women was 1.6- and 2.3-fold more likely to have LGA infants, respectively. Only excessive GWG among the normal weight group had a protective effect on SGA.

**Table 3:** Adjusted OR for birth weight classified by jointed pre-pregnancy BMI and GWG

Pre-BMI/GWG	N(%)	SGA		LGA	
		OR ^a^	95%CI	OR ^a^	95%CI
Underweight					
Insufficient	145 (4.04)	**2.961** ^b^	**1.491–5.881**	0.576	0.176–1.884
adequate	239 (6.67)	Ref.		Ref.	
Excessive	124 (3.46)	0.886	0.354–2.217	1.730	0.690–4.336
Normal weight					
Insufficient	695 (19.39)	**1.236** ^c^	**1.036–1.828**	0.988	0.699–1.397
adequate	1078 (30.07)	Ref.		Ref.	
Excessive	781 (21.79)	**0.614** ^c^	**0.388–0.972**	1.623^*^	**1.201–2.194**
Overweight/Obesity					
Insufficient	45 (1.25)	**6.672** ^c^	**1.386–32.118**	0.518	0.144–1.859
adequate	172 (4.80)	Ref.		Ref.	
Excessive	306 (8.53)	1.220	0.274–5.420	**2.287** ^c^	**1.318–3.969**

^a^ Adjusted for maternal age, occupation, gravidity, parity, GDM and gestational hypertension; ^b^ *P* < 0.001; ^c^ *P* < 0.05

The association of neonatal birth weight with GWG_rate_ under each pre-pregnancy BMI level is shown in Table 4. For women with normal BMI before pregnancy, excessive GWG_rate_ was associated with a 1.6 times higher risk for LGA but a 0.35 times lower risk for SGA. Excessive GWG_rate_ also contributed to a 1.7 times higher risk for LGA among pre-pregnancy overweight and obese women, and insufficient GWG_rate_ was associated with a 0.81 times lower LGA risk in this group.

**Table 4:** Adjusted OR for birth weight classified by jointed pre-pregnancy BMI and GWG_rate_

Pre-BMI/GWG_rate_	N(%)	SGA		LGA	
		OR ^a^	95%CI	OR ^a^	95%CI
Underweight					
Insufficient	205 (5.72)	1.760	0.799–3.874	1.860	0.556–6.220
adequate	128 (3.57)	Ref.		Ref.	
Excessive	175 (4.88)	0.910	0.370–2.239	3.305	0.990–11.038
Normal weight					
Insufficient	698 (19.47)	1.514	0.922–2.311	0.962	0.648–1.430
adequate	683 (19.05)	Ref.		Ref.	
Excessive	1173 (32.72)	**0.650** ^b^	**0.387–0.945**	**1.606** ^b^	**1.155–2.233**
Overweight/Obesity					
Insufficient	60 (1.67)	0.805	0.135–4.811	**0.190**^b^	**0.042–0.853**
adequate	128 (3.57)	Ref.		Ref.	
Excessive	335 (9.35)	0.515	0.146–1.809	**1.698** ^b^	**1.062–2.996**

^a^ Adjusted for maternal age, occupation, gravidity, parity, GDM and gestational hypertension; ^b^ *P* < 0.05

**Fig. 1:**
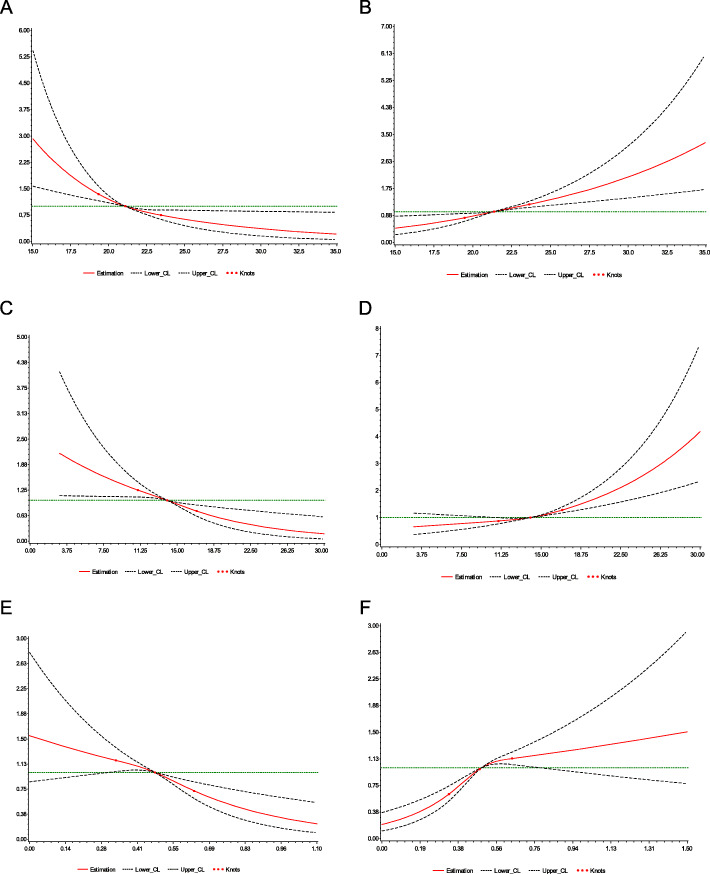
Dose-response relationships. ORs and 95%CIs for pre-pregnancy BMI (**a**,**b**), GWG (**c**,**d**) and GWG_rate_ (**e**,**f**) with SGA (**a**,**c**,**e**) and LGA (**b**,**d**,**f**). **a** and **b** adjusted for maternal age, occupation, gravidity and parity; **c**,**d**,**e**,**f** also adjusted for pre-pregnancy BMI, GDM and gestational hypertension. The solid curve in each figure is OR value while two dash curves are 95% confidence intervals

Figure 1 shows dose-response relationships between neonatal birth weight and women’s body weight before and during pregnancy. The RCS logistic regression models showed that except for the nonlinear association between GWG_rate_ and LGA (P_nonlinear_ < 0.001), the other associations were linear (P_nonlinear_ > 0.05).

## Discussion

In this study, we found that pre-pregnancy underweight and insufficient GWG were associated with higher SGA risk and lower LGA risk while pre-pregnancy overweight or obese and excessive GWG were associated with higher LGA risk and lower SGA risk. GWG_rate_ was also associated with neonatal birthweight. The association of GWG and GWG_rate_ with birth weight varies with pre-pregnancy BMI status. Dose-response relationships existed between pre-pregnancy BMI, GWG, GWG_rate_ and newborn weight.

The relationships this study found between pre-pregnancy BMI, GWG and GWG_rate_ are consistent with the findings of some existing studies [21–23], including a cohort study located in three provinces in southwest China [24]. A meta-analysis showed that insufficient GWG contributed to a lower risk for LGA while excessive GWG was associated with lower risk for SGA [11]. We also found that insufficient GWG was associated with lower LGA, but this relationship disappeared when we adjusted pre-pregnancy BMI. Xie [25] found that excessive GWG increased neonatal birth weight, but no relationship was found between pre-pregnancy BMI and birth weight. However, Ratnasiri [26] conducted a retrospective cohort study, and the results showed pre-pregnancy overweight and obese contributed to a lower SGA rate and pre-pregnancy underweight decreased the risk of LGA. These differences may be attributed to ethnicity. As for GWG_rate_, another Chinese cohort study indicated that excessive GWG_rate_ increased LGA risk, but insufficient GWG_rate_ had no impact on SGA, and this result was inconsistent with our finding [27]. Differences in findings may be due to different study population since this study was conducted in 15 different provinces and the sample could better represent Chinese women. The research mentioned above mostly focuses on specific areas of China or foreign countries, and various cultures, lifestyles, economic statuses and many other factors may contribute to the differences in the results.

The association of GWG and GWG_rate_ and birth weight varies with different pre-pregnancy BMI statuses. Stratified analysis showed that insufficient GWG increased SGA risk only in pre-pregnancy underweight or normal weight women, rather than overweight or obese women. This finding is consistent with a cohort study targeted at US women [28]. This study also found that excessive GWG decreased SGA risk only in women with a normal body weight before pregnancy. However, this finding is inconsistent with a retrospective cohort study conducted by Li [29], which showed that excessive GWG had a protective effect on SGA in pre-pregnancy overweight and obese women. We also did not find that GWG made any effort towards LGA in pre-pregnancy underweight women. Excessive GWG_rate_ was associated with a higher risk of LGA except in pre-pregnancy underweight women, and it was found to decrease SGA risk only in pre-pregnancy normal weight. However, some research [30] found that insufficient GWG_rate_ could decrease LGA risk in pre-pregnancy underweight women, but we did not find the same results. This study also did not show relationship between insufficient GWG_rate_ and the risk of SGA, which are commonly believed to be associated. This difference indicates that more comprehensive and well-designed studies are needed to help determining consistent results.

There are relatively few studies focusing on the dose-response relationship between women’s body weight before and during pregnancy and infant birth weight. Consistent results can be found through these studies that a dose-response relationship exists, but the shapes of these curves are different [31, 32]. Except for the relationship between GWG_rate_ and LGA, which showed an ‘S-shaped’ carve, others were ‘L-shaped’ in this study. These results were different from those of the previously mentioned studies, which may be attributed to different research populations or study designs. Nevertheless, dose-response research on this topic is mere, and far more studies are needed.

The novelty of this study is significant. First, this study explored the protective effects of pre-pregnancy underweight/ insufficient GWG and GWG_rate_ on LGA infants among Chinese women and the protective effects of pre-pregnancy overweight and obese/ excessive GWG and GWG_rate_ on SGA infants, which are still inconsistent yet. Second, stratified analysis was adopted to explore the relationships between GWG/GWG_rate_ and birth weight under different pre-pregnancy BMI levels, and this made our study more comprehensive and accurate; Third, this study analyzed GWG_rate_, which was less commonly researched due to cumbersome measurement, and the results can provide references for further relevant research. Fourth, we explored the dose-relationships between women’s body weight before and during pregnancy and neonatal birth weight, and this was focused by only few studies around the word.

This study has some strengths and limitations. This nationwide multicenter prospective cohort study enrolled people living in 15 different provinces in China, and this sample can well represent Chinese women. Core data, including weight before pregnancy and weight at delivery, gestational age and neonatal birth weight, were double checked to maintain data quality, which means data collected by questionnaire were checked with that recorded in the HIS system of each hospital. If there was any inconsistency, a medical record was preferred unless it was illogical. For limitations, respondents were asked to recall their pre-pregnancy body weight and height, which may result in recall bias. In addition, convenience sampling may decrease the representativeness of our results. We did not conduct subgroup analysis stratified by obesity class due to the small sample size of pre-pregnancy obese women. However, this subgroup analysis may provide more detailed references for body weight control before pregnancy and it should be explored in the future.

## Conclusions

In summary, this study showed that pre-pregnancy underweight, insufficient GWG and GWG_rate_ increased the risk of SGA while pre-pregnancy overweight or obese, excessive GWG and GWG_rate_ increased the risk of LGA. In addition, these relationships varied according to different pre-pregnancy BMI statuses. Dose-response relationships were observed between independent and dependent variates mentioned above. Our results emphasized the significance of body weight control both before and during pregnancy. Although it may be difficult for women to adhere the IOM recommendation, interventions such as education should be imposed to help achieving suitable pre-pregnancy BMI and GWG [33].

## Supplementary information

Additional file 1: Table S1. Twenty-four hospitals sites in the CPWCS project; **Table S2.** Maternal characteristics presented by pre-pregnancy BMI; **Table S3.** Maternal characteristics presented by GWG; **Table S4.** Maternal characteristics presented by GWG_rate_; **Table S5.** Birth weight classified by pre-pregnancy BMI, GWG and GWG_rate_; **Table S6.** Birth weight classified by jointed pre-pregnancy BMI and GWG; **Table S7.** Birth weight classified by jointed pre-pregnancy BMI and GWG_rate_. (DOCX 53 kb)

## Data Availability

The datasets used and/or analyzed during the current study are available from the corresponding author on reasonable request.

## Abbreviations

BMI: Body mass index
GWG: Gestational weight gain
GWG_rate_: Rate of gestational weight gain during the second and third trimesters
SGA: Small for gestational age
LGA: Large for gestational age
