# Supplementary material for: Association among pre-pregnancy body mass index, gestational weight gain and neonatal birth weight: a prospective cohort study in China
Source: BMC Pregnancy Childbirth. 2020 Nov 12;20:690. doi: 10.1186/s12884-020-03323-x (PMC7659120; doi:10.1186/s12884-020-03323-x)
Supplement: Supplementary file 1 — Twenty-four hospitals sites in the CPWCS project; Table S2. Maternal characteristics presented by pre-pregnancy BMI; Table S3. Maternal characteristics presented by GWG; Table S4. Maternal characteristics presented by GWGrate; Table S5. Birth weight classified by pre-pregnancy BMI, GWG and GWGrate; Table S6. Birth weight classified by jointed pre-pregnancy BMI and GWG; Table S7. Birth weight classified by jointed pre-pregnancy BMI and GWGrate. (DOCX 53 kb) [file 12884_2020_3323_MOESM1_ESM.docx]

Table S1 Twenty-four hospitals sites in the CPWCS project

| Code | Hospital name | Location | Region 1^a^ | Region 2^b^ |
| --- | --- | --- | --- | --- |
| 1 | Maternity and Children’s Health Care Centers in Urumchi | Urumchi, Xinjiang | North | West |
| 2 | Guiyang Maternity and Child Health Hospital | Guiyang, Guizhou | South | West |
| 3 | Northwest Women and Children’s Hospital | Xi’an, Shaanxi | North | West |
| 4 | Changsha Hospital for Maternal and Child Health Care | Changsha, Hunan | South | Central |
| 5 | The Third Affiliated Hospital of Zhengzhou University | Zhengzhou, Henan | North | Central |
| 6 | Jiaxian Maternal and Child Health Care Hospital | Jiaxian, Henan | North | Central |
| 7 | Changzhou No.2 People’s Hospital | Changzhou, Jiangsu | South | East |
| 8 | Maternal and Child Health Care Hospital of Jin’an District | Liu’an, Anhui | South | Central |
| 9 | People′s Hospital in Dong′e County of Shandong Province | Dong’e, Shandong | North | East |
| 10 | Dongguan Maternal and Child Health Care Hospital | Dongguan, Guangdong | South | East |
| 11 | Bayannur Linhe District Maternal and Child Health Hospital | Bayannur, Inner Mongolia | North | West |
| 12 | Affiliated Hospital of Guizhou Medical University | Guiyang, Guizhou | South | West |
| 13 | Yangzhou Maternal and Child Health Hospital | Yangzhou, Jiangsu | South | East |
| 14 | Affiliated Hospital of Jining Medical University | Jining, Shandong | North | East |
| 15 | Zaozhuang maternal and child health hospital | Zaozhuang, Shandong | North | East |
| 16 | China-Japan Friendship Hospital Affiliated Jilin University | Changchun, Jilin | North | Central |
| 17 | Affiliated hospital of Jiujiang Medical college | Jiujiang, Jiangxi | South | Central |
| 18 | The First Affiliated Hospital of Nanchang University | Nanchang, Jiangxi | South | Central |
| 19 | Chengdu Women’s and Children’s Central Hospital | Chengdu, Sichuan | South | West |
| 20 | the Central Hospital of Three Gorges of Chongqing | Wanzhou, Chongqing | South | West |
| 21 | Shaanxi Provincial People’s Hospital | Xi’an, Shaanxi | North | West |
| 22 | Xingyang Maternal and Child Health Hospital | Zhengzhou, Henan | North | Central |
| 23 | Changchun Obstetrics-Gynecology Hospital | Changchun, Jilin | North | Central |
| 24 | Tongzhou Maternal and Child Health Hospital of Beijing | Tongzhou, Beijing | North | East |

^a^ Region that participants living in was categorized into North and South China according to the Qinling-Huaihe Line in China.

^b^ Region that participants living in was categorized into East, Central and West China according to the Chinese Health Statistics Yearbook.

Table S2 Maternal characteristics presented by pre-pregnancy BMI [n(%)]

| Items | Underweight | Normal weight | overweight | obesity | χ^2^/H | P |
| --- | --- | --- | --- | --- | --- | --- |
|  | N=508 | N=2554 | N=461 | N=62 |  |  |
| Age |  |  |  |  | 80.088 | **<0.001** |
| <25 | 120(23.62) | 387(15.15) | 62(13.45) | 7(11.29) |  |  |
| 25~ | 292(57.48) | 1266(49.57) | 213(46.20) | 33(53.23) |  |  |
| 30~ | 81(15.95) | 643(25.18) | 120(26.03) | 15(24.19) |  |  |
| 35~ | 15(2.95) | 258(10.10) | 66(14.32) | 7(11.29) |  |  |
| Ethnicity |  |  |  |  | 4.204 | 0.240 |
| Han | 481(94.67) | 2429(95.11) | 446(96.75) | 57(91.94) |  |  |
| Minority | 27(5.33) | 125(4.89) | 15(3.35) | 5(8.06) |  |  |
| Residential Areas |  |  |  |  | 13.567 | **0.004** |
| Urban | 195(38.39) | 1173(45.93) | 206(44.69) | 20(32.26) |  |  |
| Rural | 313(61.61) | 1381(54.07) | 255(55.31) | 42(67.73) |  |  |
| Educational level |  |  |  |  | 44.287 | **<0.001** |
| Less than high school | 65(12.80) | 335(13.12) | 93(20.17) | 15(24.20) |  |  |
| High school | 105(20.66) | 532(20.83) | 104(22.56) | 24(38.71) |  |  |
| Bachelor | 305(60.04) | 1509(59.08) | 246(53.36) | 322(35.48) |  |  |
| Master or above | 33(6.50) | 178(6.97) | 18(3.91) | 1(1.61) |  |  |
| Occupation |  |  |  |  | 26.593 | **0.002** |
| No | 169(33.27) | 709(27.76) | 157(34.06) | 29(46.77) |  |  |
| Yes | 339(66.73) | 1845(72.24) | 304(65.94) | 33(53.23) |  |  |
| Annual household Income(thousand) | |  |  |  | 49.090 | **<0.001** |
| <70 | 123(24.21) | 650(25.45) | 153(33.19) | 33(53.23) |  |  |
| 70~ | 228(44.88) | 1186(46.44) | 218(47.29) | 24(38.71) |  |  |
| 200~ | 157(30.91) | 718(28.11) | 90(19.52) | 5(8.06) |  |  |
| Smoking |  |  |  |  | 0.770 | 0.857 |
| No | 482(94.88) | 2444(95.69) | 439(95.23) | 59(95.16) |  |  |
| Yes | 26(5.12) | 110(4.31) | 22(4.77) | 3(4.84) |  |  |
| Alcohol consumption | |  |  |  | 1.057 | 0.788 |
| No | 475(93.50) | 2368(92.72) | 426(92.41) | 56(90.32) |  |  |
| Yes | 33(6.50) | 186(7.28) | 35(7.39) | 6(9.68) |  |  |
| Gravidity |  |  |  |  | 60.314 | **<0.001** |
| 0 | 185(36.41) | 785(30.74) | 106(23.00) | 9(14.52) |  |  |
| 1 | 192(37.80) | 830(32.50) | 147(31.89) | 23(37.09) |  |  |
| 2 | 80(15.75) | 555(21.73) | 107(23.21) | 13(20.97) |  |  |
| ≥3 | 51(10.04) | 384(15.03) | 101(21.90) | 17(27.42) |  |  |
| Parity |  |  |  |  | 35.16 | **<0.001** |
| 0 | 343(67.52) | 1460(57.17) | 243(52.71) | 34(54.84) |  |  |
| 1 | 142(27.95) | 946(37.04) | 184(39.92) | 20(32.26) |  |  |
| 2 | 16(3.15) | 97(3.80) | 25(5.42) | 7(11.29) |  |  |
| ≥3 | 7(1.38) | 51(1.99) | 9(1.95) | 1(1.61) |  |  |
| GMD |  |  |  |  | 14.995 | **0.002** |
| No | 459(90.35) | 2201(86.18) | 378(82.00) | 51(82.26) |  |  |
| Yes | 49(9.65) | 353(13.82) | 83(18.00) | 11(17.74) |  |  |
| Gestational Hypertension | |  |  |  | 53.979 | **<0.001** |
| No | 503(99.02) | 2490(97.49) | 427(92.62) | 55(88.71) |  |  |
| Yes | 5(0.98) | 64(2.51) | 34(7.38) | 7(11.29) |  |  |
| Gestational Week | 39.15±1.58 | 39.27±1.40 | 39.16±1.51 | 39.06±1.38 | 5.250 | 0.154 |

Table S3 Maternal characteristics presented by GWG [n(%)]

| Items | GWG | | |  | |  | |
| --- | --- | --- | --- | --- | --- | --- | --- |
|  | Insufficient N=885 | Sufficient N=1489 | Excessive N=1211 | χ^2^/H | P | |  |
| Age |  |  |  | 12.639 | **0.049** | |  |
| <25 | 140(15.82) | 259(12.39) | 177(14.61) |  |  | |  |
| 25~ | 430(48.59) | 719(48.29) | 655(54.09) |  |  | |  |
| 30~ | 218(24.63) | 370(24.85) | 271(22.38) |  |  | |  |
| 35~ | 97(10.96) | 141(9.47) | 108(8.92) |  |  | |  |
| Ethnicity |  |  |  | 1.058 | 0.589 | |  |
| Han | 848(95.82) | 1413(94.90) | 1152(95.13) |  |  | |  |
| Minority | 37(4.18) | 76(5.10) | 59(4.87) |  |  | |  |
| Residential Areas |  |  |  | 3.431 | 0.180 | |  |
| Urban | 416(47.01) | 642(43.12) | 536(44.26) |  |  | |  |
| Rural | 469(52.99) | 847(56.88) | 675(55.74) |  |  | |  |
| Educational level |  |  |  | 5.384 | 0.496 | |  |
| Less than high school | 122(13.79) | 218(14.64) | 168(13.87) |  |  | |  |
| High school | 185(20.90) | 316(21.22) | 264(21.80) |  |  | |  |
| Bachelor | 517(58.42) | 849(57.02) | 716(59.13) |  |  | |  |
| Master or above | 61(6.89) | 106(7.12) | 63(5.20) |  |  | |  |
| Occupation |  |  |  | 1.425 | 0.964 | |  |
| No | 266(30.06) | 442(29.68) | 356(29.40) |  |  | |  |
| Yes | 619(69.94) | 1047(70.32) | 855(70.60) |  |  | |  |
| Annual household Income(thousand) | |  |  | 7.610 | 0.107 | |  |
| <70 | 226(25.54) | 377(25.32) | 356(29.40) |  |  | |  |
| 70~ | 405(45.76) | 710(47.68) | 541(44.67) |  |  | |  |
| 200~ | 254(28.70) | 402(27.00) | 314(25.93) |  |  | |  |
| Smoking |  |  |  | 0.355 | 0.837 | |  |
| No | 848(95.82) | 1419(95.30) | 1157(95.54) |  |  | |  |
| Yes | 37(4.18) | 70(4.70) | 54(4.46) |  |  | |  |
| Alcohol consumption |  |  |  | 1.664 | 0.435 | |  |
| No | 813(91.86) | 1389(93.28) | 1123(92.73) |  |  | |  |
| Yes | 72(8.14) | 100(6.72) | 88(7.27) |  |  | |  |
| Gravidity |  |  |  | 4.998 | 0.544 | |  |
| 0 | 281(31.75) | 460(30.89) | 344(28.41) |  |  | |  |
| 1 | 284(32.09) | 495(33.24) | 413(34.10) |  |  | |  |
| 2 | 189(21.36) | 297(19.95) | 269(22.21) |  |  | |  |
| ≥3 | 131(14.80) | 237(15.92) | 185(15.28) |  |  | |  |
| Parity |  |  |  | 14.910 | **0.021** | |  |
| 0 | 472(53.33) | 871(58.50) | 737(60.86) |  |  | |  |
| 1 | 349(39.44) | 533(35.80) | 410(33.86) |  |  | |  |
| 2 | 40(4.52) | 58(3.89) | 47(3.88) |  |  | |  |
| ≥3 | 24(2.71) | 27(1.81) | 17(1.40) |  |  | |  |
| GMD |  |  |  | 21.255 | **<0.001** | |  |
| No | 725(81.92) | 1287(86.43) | 1077(88.94) |  |  | |  |
| Yes | 160(18.08) | 202(13.57) | 134(11.06) |  |  | |  |
| Gestational Hypertension | |  |  | 14.091 | **0.001** | |  |
| No | 868(98.08) | 1451(97.45) | 1156(95.46) |  |  | |  |
| Yes | 17(1.92) | 38(2.55) | 55(4.54) |  |  | |  |
| Gestational Week | 38.97±1.63 | 39.03±1.33 | 39.26±1.41 | 36.739 | **<0.001** | |  |

Table S4 Maternal characteristics presented by GWG_rate_ [n(%)]

| Items | GWG_rate_ | | |  | |  | |
| --- | --- | --- | --- | --- | --- | --- | --- |
|  | Insufficient N=963 | Sufficient N=939 | Excessive N=1683 | χ^2^/H | P | |  |
| Age |  |  |  | 13.941 | **0.030** | |  |
| <25 | 138(14.33) | 154(16.40) | 284(16.87) |  |  | |  |
| 25~ | 463(48.08) | 465(49.52) | 876(52.05) |  |  | |  |
| 30~ | 251(26.06) | 235(25.03) | 373(22.16) |  |  | |  |
| 35~ | 111(11.53) | 85(9.05) | 150(8.92) |  |  | |  |
| Ethnicity |  |  |  | 3.596 | 0.166 | |  |
| Han | 913(94.81) | 886(94.36) | 1614(95.90) |  |  | |  |
| Minority | 50(5.19) | 53(5.64) | 69(4.10) |  |  | |  |
| Residential Areas |  |  |  | 1.611 | 0.447 | |  |
| Urban | 443(46.00) | 405(43.13) | 746(44.33) |  |  | |  |
| Rural | 520(54.00) | 534(56.87) | 973(55.67) |  |  | |  |
| Educational level |  |  |  | 11.312 | 0.079 | |  |
| Less than high school | 122(12.67) | 119(12.67) | 267(15.86) |  |  | |  |
| High school | 223(23.16) | 205(21.83) | 337(20.03) |  |  | |  |
| Bachelor | 562(58.36) | 546(58.15) | 974(57.87) |  |  | |  |
| Master or above | 56(5.81) | 69(7.35) | 105(6.24) |  |  | |  |
| Occupation |  |  |  | 7.431 | 0.283 | |  |
| No | 277(28.76) | 253(26.95) | 534(31.73) |  |  | |  |
| Yes | 686(71.24) | 686(73.05) | 1149(68.27) |  |  | |  |
| Annual household Income(thousand) | |  |  | 1.227 | 0.874 | |  |
| <70 | 259(26.90) | 239(25.45) | 461(27.39) |  |  | |  |
| 70~ | 444(46.11) | 439(46.75) | 773(45.93) |  |  | |  |
| 200~ | 260(26.99) | 261(27.80) | 449(26.68) |  |  | |  |
| Smoking |  |  |  | 2.736 | 0.255 | |  |
| No | 912(94.70) | 904(96.27) | 1608(95.54) |  |  | |  |
| Yes | 51(5.30) | 35(3.73) | 75(4.46) |  |  | |  |
| Alcohol consumption |  |  |  | 0.071 | 0.965 | |  |
| No | 892(92.63) | 870(92.65) | 1563(92.87) |  |  | |  |
| Yes | 71(7.37) | 69(7.35) | 120(7.13) |  |  | |  |
| Gravidity |  |  |  | 3.783 | 0.706 | |  |
| 0 | 282(29.28) | 288(30.67) | 515(30.60) |  |  | |  |
| 1 | 317(32.92) | 310(33.01) | 565(33.57) |  |  | |  |
| 2 | 209(21.70) | 209(22.26) | 337(20.02) |  |  | |  |
| ≥3 | 155(16.10) | 132(14.06) | 266(15.81) |  |  | |  |
| Parity |  |  |  | 12.070 | 0.060 | |  |
| 0 | 525(54.52) | 537(57.19) | 1018(60.49) |  |  | |  |
| 1 | 374(38.83) | 340(36.21) | 578(34.34) |  |  | |  |
| 2 | 43(4.47) | 39(4.15) | 63(3.74) |  |  | |  |
| ≥3 | 21(2.18) | 23(2.45) | 24(1.43) |  |  | |  |
| GMD |  |  |  | 33.952 | **<0.001** | |  |
| No | 783(81.31) | 802(85.41) | 1504(89.36) |  |  | |  |
| Yes | 180(18.69) | 137(14.59) | 179(10.64) |  |  | |  |
| Gestational Hypertension | |  |  | 3.409 | 0.182 | |  |
| No | 941(97.72) | 911(97.02) | 1623(96.43) |  |  | |  |
| Yes | 22(2.28) | 28(3.98) | 60(3.57) |  |  | |  |
| Gestational Week | 39.15±1.58 | 39.36±1.27 | 39.20±1.45 | 36.739 | <0.001 | |  |

Table S5 Birth weight classified by pre-pregnancy BMI, GWG and GWG_rate_ [n(%)]

| Pre-BMI  /GWG | Average Birth Weight(g) | N  (n=3585) | Birth Weight Groups | | | χ^2^ | P |
| --- | --- | --- | --- | --- | --- | --- | --- |
|  |  |  | SGA  (n=207) | AGA  (n=3000) | LGA  (n=378) |  |  |
| Pre-BMI |  |  |  |  |  | 75.851 | **<0.001** |
| Underweight | 3186±465 | 508(14.17) | 52(25.12) | 428(14.27) | 28(7.41) |  |  |
| Normal weight | 3325±439 | 2554(71.24) | 141(68.12) | 2160(72.00) | 253(66.93) |  |  |
| Overweight | 3435±488 | 461(12.86) | 13(6.28) | 359(11.97) | 89(23.54) |  |  |
| Obesity | 3416±378 | 62(1.73) | 1(0.48) | 53(1.76) | 8(2.12) |  |  |
| GWG |  |  |  |  |  |  |  |
| Insufficient | 3196±464 | 885(24.69) | 81(39.13) | 738(24.60) | 66(17.46) | 69.826 | **<0.001** |
| adequate | 3318±423 | 1489(41.53) | 84(40.58) | 1278(42.60) | 127(33.60) |  |  |
| Excessive | 3416±458 | 1211(33.78) | 42(20.29) | 984(32.80) | 185(48.95) |  |  |
| GWG_rate_ |  |  |  |  |  | 66.353 | **<0.001** |
| Insufficient | 3251±480 | 963(26.86) | 89(43.00) | 808(26.93) | 66(17.46) |  |  |
| adequate | 3328±409 | 939(26.19) | 56(27.05) | 803(26.77) | 80(21.16) |  |  |
| Excessive | 3358±456 | 1683(46.95) | 62(29.95) | 1389(46.30) | 232(61.38) |  |  |

Table S6 Birth weight classified by jointed pre-pregnancy BMI and GWG [n(%)]

| Pre-BMI  /GWG | N | SGA  (N=207) | AGA  (N=3000) | LGA  (N=378) | χ^2^ | P |
| --- | --- | --- | --- | --- | --- | --- |
| Underweight |  |  |  |  | 19.344 | **0.001** |
| Insufficient | 145(4.04) | 27(13.04) | 114(3.80) | 4(1.06) |  |  |
| adequate | 239(6.67) | 17(8.21) | 209(6.97) | 13(3.44) |  |  |
| Excessive | 124(3.46) | 8(3.86) | 105(3.50) | 11(2.91) |  |  |
| Normal weight |  |  |  |  | 20.138 | **<0.001** |
| Insufficient | 695(19.39) | 49(23.67) | 587(19.57) | 59(15.61) |  |  |
| adequate | 1078(30.07) | 64(30.92) | 922(30.73) | 92(24.34) |  |  |
| Excessive | 781(21.79) | 28(13.53) | 651(21.70) | 102(26.98) |  |  |
| Overweight/Obesity |  |  |  |  | 25.449 | **<0.001** |
| Insufficient | 45(1.25) | 5(2.42) | 37(1.23) | 3(0.79) |  |  |
| adequate | 172(4.80) | 3(1.45) | 147(4.90) | 22(5.82) |  |  |
| Excessive | 306(8.53) | 6(2.90) | 228(7.60) | 72(19.05) |  |  |

Table S7 Birth weight classified by jointed pre-pregnancy BMI and GWG_rate_ [n(%)]

| Pre-BMI  /GWG_rate_ | N | SGA  (N=207) | AGA  (N=3000) | LGA  (N=378) | χ^2^ | P |
| --- | --- | --- | --- | --- | --- | --- |
| Underweight |  |  |  |  | 7.583 | **0.048** |
| Insufficient | 205(5.72) | 29(14.01) | 166(5.53) | 10(2.65) |  |  |
| adequate | 128(3.57) | 10(4.83) | 113(3.77) | 5(1.32) |  |  |
| Excessive | 175(4.88) | 13(6.28) | 149(4.97) | 13(3.44) |  |  |
| Normal weight |  |  |  |  | 30.665 | **<0.001** |
| Insufficient | 698(19.47) | 58(28.02) | 586(19.53) | 54(14.29) |  |  |
| adequate | 683(19.05) | 41(19.81) | 587(19.57) | 55(14.55) |  |  |
| Excessive | 1173(32.72) | 42(20.29) | 987(32.90) | 144(38.09) |  |  |
| Overweight/Obesity |  |  |  |  | 14.104 | **0.007** |
| Insufficient | 60(1.67) | 2(0.97) | 56(1.87) | 2(0.53) |  |  |
| adequate | 128(3.57) | 5(2.41) | 103(3.43) | 20(5.29) |  |  |
| Excessive | 335(9.35) | 7(3.38) | 253(8.43) | 75(19.84) |  |  |
